# Incidental detection of Cladosporium in cytology

**DOI:** 10.1186/s13000-024-01469-2

**Published:** 2024-02-29

**Authors:** Tummidi Santosh, Indranil Chakrabarti, Aparna Palit, Sonakshi Srivastava

**Affiliations:** 1 Department of Pathology & Lab Medicine, AIIMS, Kalyani, West Bengal India; 2 Department of Dermatology & Venereology, AIIMS, Kalyani, West Bengal India; 3 Department of Microbiology, AIIMS, Kalyani, West Bengal India

**Keywords:** Cladosporium, Culture, Cytology, Fungal

## Abstract

**Background:**

Fungal infection incidental detection is a common encounter in cytopathology practices. Detection of the fungal organisms and awareness of the morphological features are challenges for the cytopathologist.

**Case presentation:**

We report a case of incidental detection of a fungal organism in a 67-year-old male patient with complaints of bilateral elbow joint swellings. Cytology was done and showed a fungal organism (Cladosporium sps.).

**Conclusion:**

Fine needle aspiration cytology (FNAC) along with Rapid on-site evaluation (ROSE) is a rapid, minimally invasive technique used for the diagnosis and detection of various fungi / parasites leading to early and definitive treatment.

**Supplementary Information:**

The online version contains supplementary material available at 10.1186/s13000-024-01469-2.

## Background

Fine needle aspiration (FNA) is an easy and cost-effective technique to diagnose various neoplastic and non-neoplastic conditions [1]. Detection of fungal inflammatory lesions has been on the rise due to altered immunity in the form of chemotherapy treatments, HIV, Diabetics mellitus, etc. [1]. Cytological samples from exfoliative sampling or FNAC procedures can be used for microorganisms and /or their cytopathologic effect caused by the infection. Detection of fastidious organisms such as fungal and mycobacterial organisms is easier to detect on cytology compared to conventional culture or microbiological techniques [2]. We report a case of Cladosporium sps incidentally detected on the cytological sample and later confirmed by culture.

## Case presentation

**Fig. 1:**
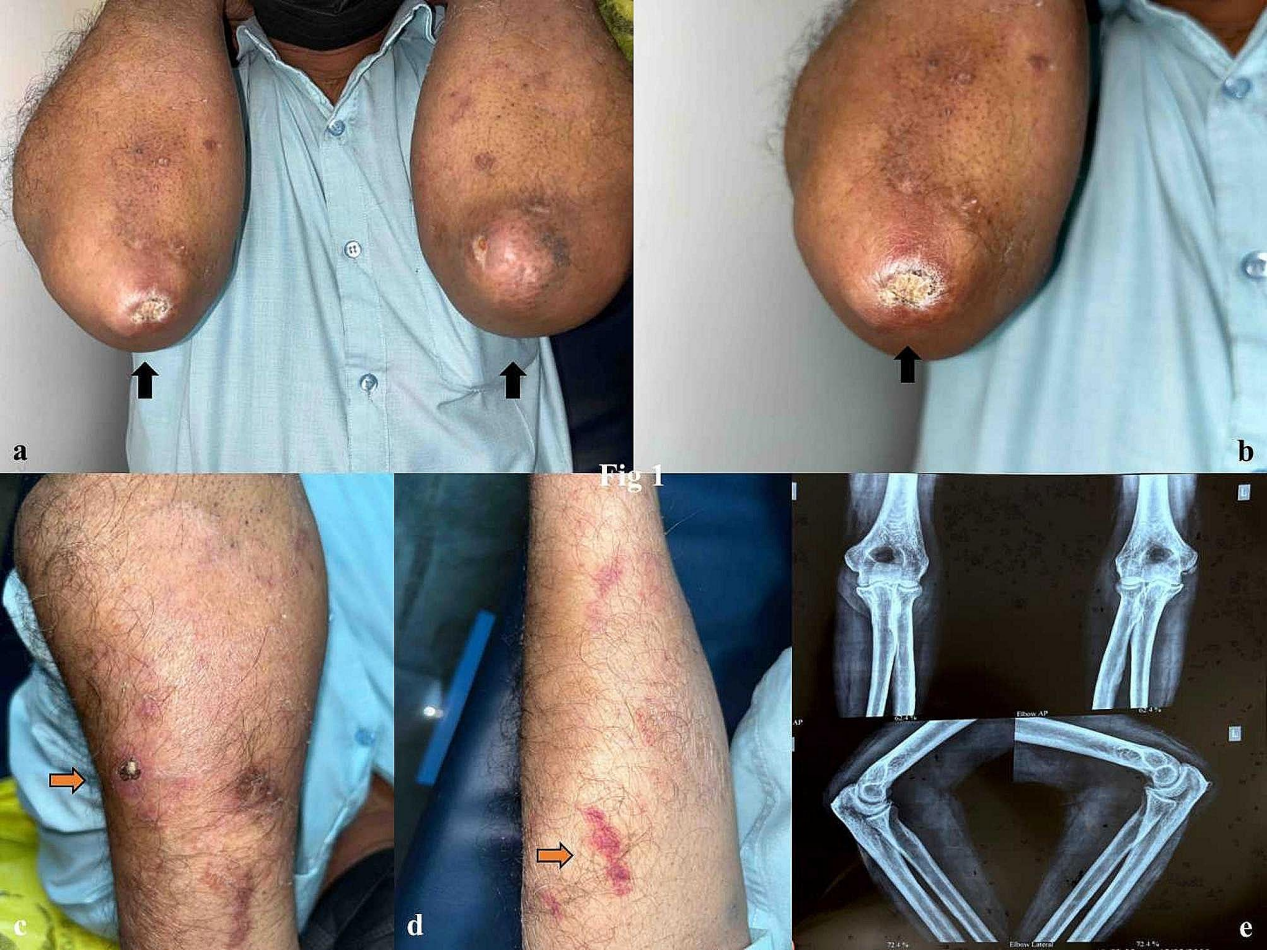
**a,b,c,d**: Swelling in the right elbow posterior aspect measuring 2 × 2 cm, firm to hard and non-tender. Another swelling in the left elbow is firm to hard with overlying reddish skin. There were a few purpuric lesions on the arms with superficial crusting. **e**: X-ray from B/L elbow shows inflammation with irregular fluid collection into the posterior aspect of the right elbow in the subcutaneous plane and irregular erosion of the olecranon

A 67-year-old male patient presented to our dermatology outpatient department with complaints of bilateral joint pain along with a few senile purpuric patches over the arms. On examination, there was a swelling in the right elbow posterior aspect measuring 2 × 2 cm, firm to hard and non-tender on palpation with the presence of ulceration for 3 months. Another swelling in the left elbow is firm to hard with overlying reddish skin present for the last 1 year. [Fig. 1a,b,c,d] The patient had a history of falls 6 months back over bilateral elbows. There were a few purpuric lesions on the arms with superficial crusting.

X-ray from B/L elbow shows inflammation with irregular fluid collection into the posterior aspect of the right elbow in the subcutaneous plane and irregular erosion of the olecranon. Features were suggestive of infective/inflammatory etiology. [Fig. 1e]

Routine laboratory investigations revealed Haemoglobin- 13.9gm/dl, Total leucocyte count-14.3*10^3^/µL; Fasting sugar- 261 mg/dl, Postprandial sugar- 227 mg/dl, and HbA1c- 8.2%. Lipid profile revealed higher triglycerides values (203 mg/dl), and other parameters were normal. His viral serological markers were negative. Ziehl-Neelsen (ZN) stain for Acid fast bacilli (AFB) in the smear was negative & Catridge based Nuclei acid amplification testing (CB-NAAT) was negative from the sputum sample. With the above findings, the clinical suspicion was of gouty arthritis.

**Fig. 2:**
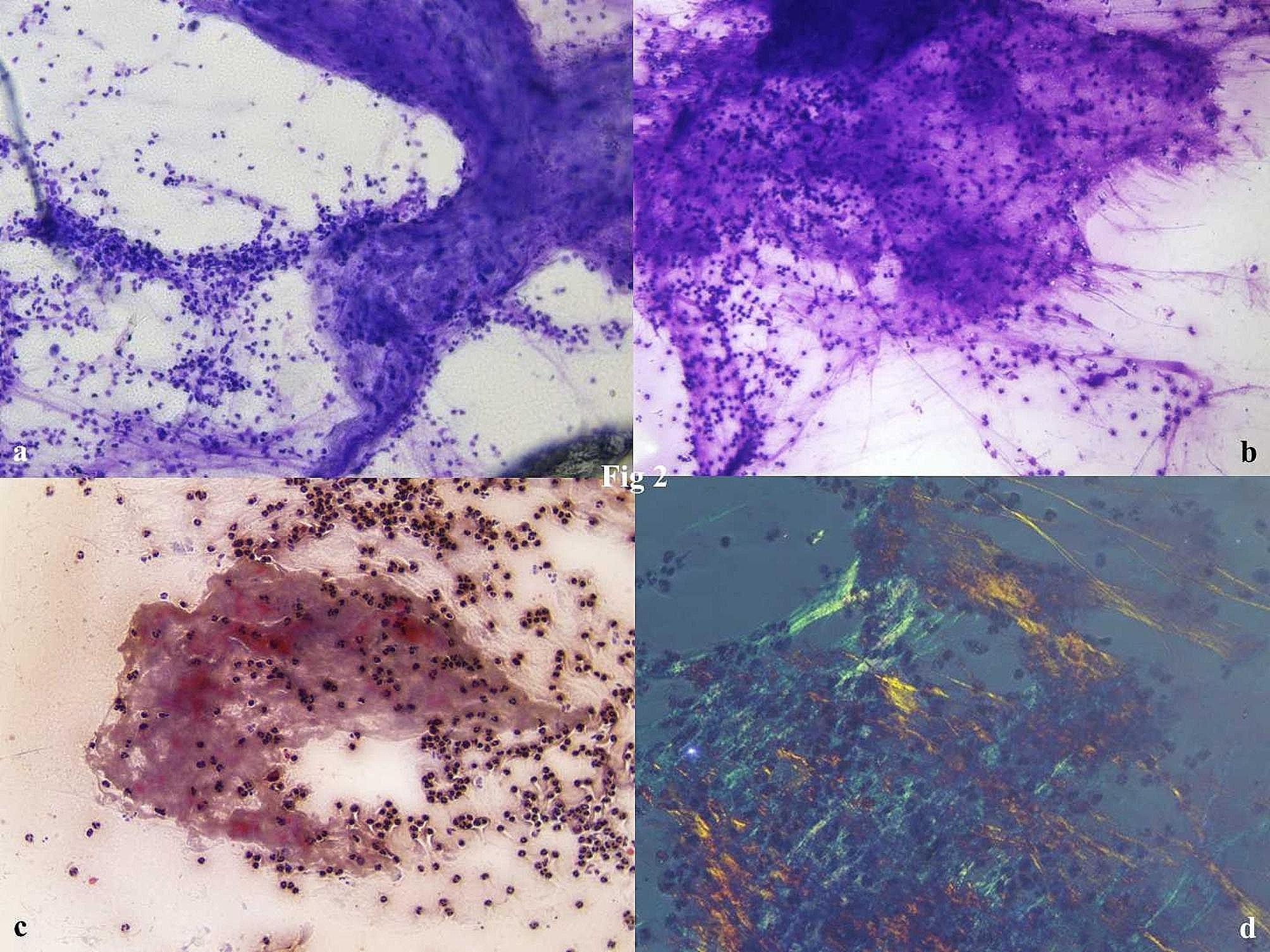
**a,b,c**: Cytosmears revealed the presence of numerous neutrophils with fibro histiocytic tangles, cyst macrophages, nuclear debris, and necrotic granular fluid background. [Tol Blue, x40; Geimsa, x40; PAP, x40] **d**: Polarising microscopy shows the absence of any crystals. [Pol, x40]

**Fig. 3:**
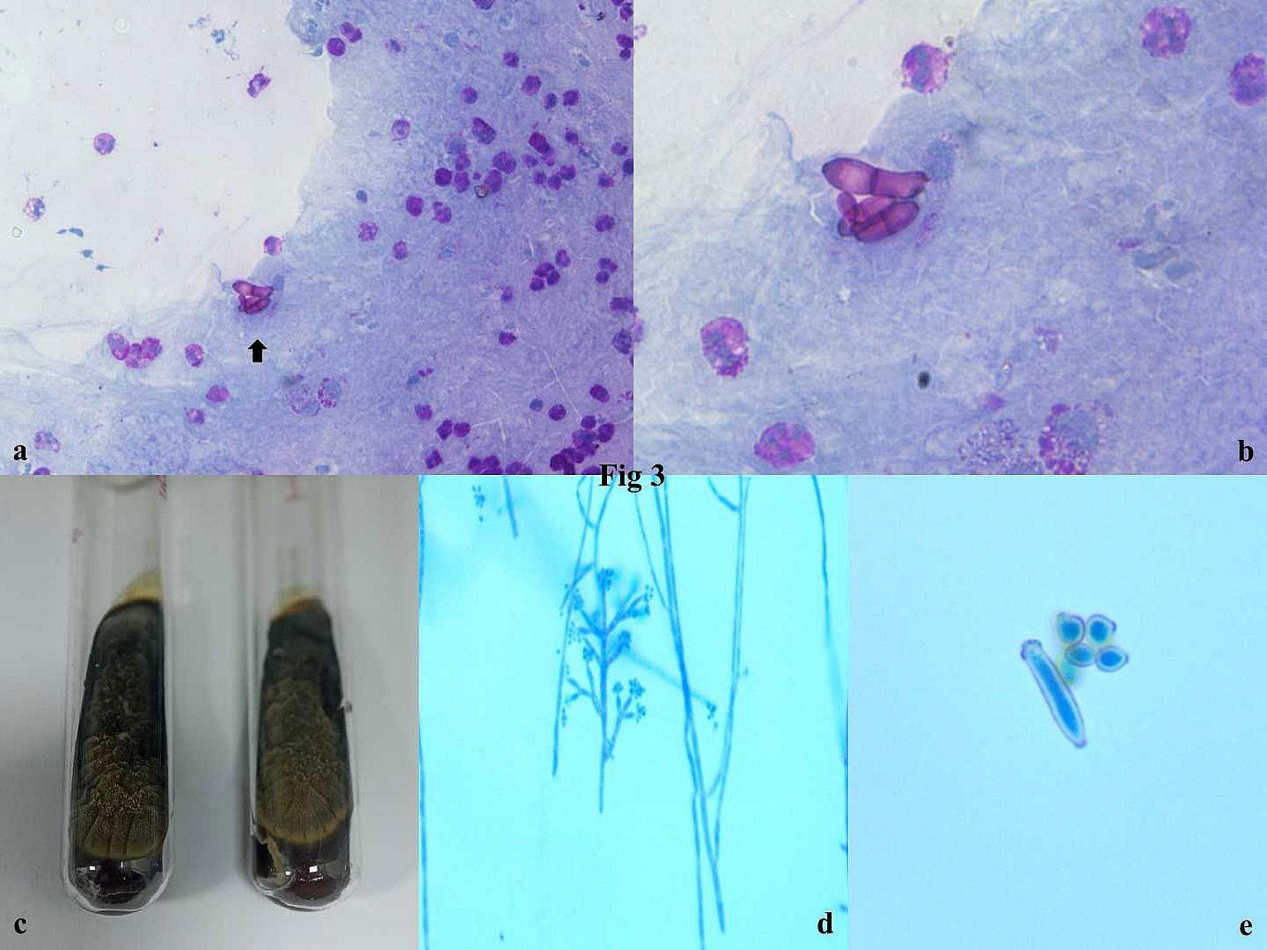
**a,b**: Special stain with periodic acid schiff showed fungal spores. **c,d,e**: Culture tube showing an melanized pigmented fungi and also in Lactophenol cotton blue staining [PAS, x100; 40,x SDA]

The patient was advised FNAC from the swelling in bilateral elbows. Rapid on-site evaluation (ROSE) was performed using 1% aq. toluidine blue. There was 1 ml of pus-like material aspirated from the left lesion and 1 ml of straw yellow fluid from the right lesion. Smears in ROSE revealed the presence of numerous neutrophils with fibro histiocytic tangles. The slides from both elbows were labeled separately and sent to routine cytological staining, MGG and PAP smears for the slide showed plenty of neutrophils, cyst macrophages, nuclear debris, and necrotic granular fluid background. There was the presence of a few fungal spores in the left elbow smears. A Periodic-acid Schiff (PAS) stain was done and confirmed the fungal spores. [Figures 2a-d & 3a, b] ZN stain of smears was negative for mycobacterium bacilli. The polarised microscope revealed no crystals. The separate sterile sample was then sent for fungal culture with a positive growth after 21 days morphologically resembling Cladosporium sps. [Fig. 3] The patient was called for revaluation and was advised itraconazole in a dosage schedule of 200 mg BD for 1 month. There is gradual decrease in size of the lesions and the patient has been kept under follow up.

## Discussion

The ability to detect, diagnose infections and inflammatory lesions is an essential part of cytopathology services [3]. The cytological sample acquired through minimally invasive techniques is preferred for early diagnosis and management of patients. Sample from exfoliative sites (buccal, cervical, sputum, BAL) or abrasive (such as brushings, EUS, washings, and lavages) and FNAC from superficial and deep-seated lesions can be used for rapid staining of tissue to do the ancillary studies [2–4]. Given the fact that fastidious organism takes a long time for growth on culture media, cytological evaluation and looking for characteristic morphological features can play a vital role in timely treatment and further management [5]. 

Phaeohyphomycosis is a chronic cutaneous, subcutaneous, and systemic mycotic infection caused by dematiaceous fungi, which include the genera Alternaria, Curvularia, Phialophora, Exophiala, and Cladosporium [4, 6]. 

Cladosporium sps is widely distributed, occurring as common saprophytes in soil [5]. The risk factors for Cladosporium infection include injury to exposed parts of the body, diabetes mellitus, organ transplantation, autoimmune diseases, human immunodeficiency virus, Mycobacterium tuberculosis, etc. Trauma accounts for the most common cause of cerebral, pulmonary, pancreatic, ocular, nail, and mostly subcutaneous infections. Clinically the lesions present as papules, nodules, and cysts located in the extremities [6]. Typical lesions of subcutaneous phaeohyphomycosis present as nodules or abscesses with the tendency of lymphatic or hematogenous dissemination [7]. Cladosporium and its spores have been reported to cause respiratory allergy i.e. asthma, or pulmonary infection, chronic rhinosinusitis, Alzheimer’s disease [8]. 

FNAC with May-Grunwald-Giemsa and Papanicolaou stains help suspect necro-inflammatory with Splendore-Hoeppli phenomenon, neutrophils, degenerated cells, granulomatous response, and acute angle branched hyphae of 2–6 μm with close septations, terminal bulbous vesicular swellings, and yeast-like forms are diagnostic. Periodic Acid Schiff’s stains the fungal organisms red/pink and nuclei blue, and Grocott methenamine silver (GMS) stains the fungal organisms black with a background green stain. However, GMS has limitations of a high degree of background staining sometimes leading to difficulty in the appreciation of morphologic features. Masson Fontana can highlight the melanized fungi [4, 5, 9]. 

Molecular tools can be used for species identification with limited availability. Surgical excision and drainage of the lesion remain the main stay with or without anti-fungal therapy. Antifungals such as amphotericin, fluconazole, ketoconazole, terbinafine and 5-flurocytosine (5FC) may be used for patients with severe disease, poor response or hepatic toxicity to itraconazole [4, 10]. 

## Conclusion

Use of FNAC with onsite evaluation can be helpful for real-time screening of samples and triage of samples for diagnostic workup in the form of the cell block, culture, or molecular techniques. Routine stain and clinical differentials of fungal infection in case of injury history can add to early diagnosis and treatment.

### Electronic supplementary material

Below is the link to the electronic supplementary material.

Supplementary Material 1

## Data Availability

All the data regarding the findings are available within the manuscript.

## Abbreviations

5FC: 5-flurocytosine
AFB: Acid fast Bacilli
CB-NAAT: Catridge based nucleic acid amplification test
FNAC: Fine needle aspiration cytology
GMS: Grocott methenamine silver
PAP: Papanicolaou
PAS: Periodic acid schiff
ROSE: Rapid On-site evaluation
ZN: Ziehl neelsen
