# Supplementary material for: Incidental detection of Cladosporium in cytology
Source: Diagn Pathol. 2024 Feb 29;19:45. doi: 10.1186/s13000-024-01469-2 (PMC10903045; doi:10.1186/s13000-024-01469-2)
Supplement: Supplementary file 1 — Supplementary Material 1 [file 13000_2024_1469_MOESM1_ESM.docx]

| **Title of Article** | **Incidental detection of Cladosporium Fungal Organism in cytology** | |
| --- | --- | --- |
| **Running title** | *Incidental detection of Cladosporium* | |
| **Article Type** | **Case report** | |
| **S no.** | **Author** | **Details** |
| 1 | **Corresponding Author:** | **Dr Tummidi Santosh**  Associate professor  Department of Pathology & Lab Medicine  AIIMS, Kalyani, W.B.,  e-mail: born_vss@yahoo.co.in  Phn- 08895495670 |
| 2 | Co-author: | Dr Indranil Chakrabarti  Additional Professor  Department of Pathology & Lab Medicine  AIIMS, Kalyani, W.B.,  e-mail: indranil.patho@aiimskalyani.edu.in |
| 3 | Co-author: | Dr Aparna Palit  Prof. & HOD  Department of Dermatology  AIIMS, Kalyani, W.B.,  email: [aparna.derm@aiimskalyani.edu.in](mailto:aparna.derm@aiimskalyani.edu.in) |
| 4 | Co-author: | Dr Sonakshi Srivastava  Assistant professor  Department of Microbiology  AIIMS, Kalyani, W.B  e-mail: sonakshi.micro@aiimskalyani.edu.in |

**Conflict of interest: Nil**

**Disclosure of grants: Nil**
